# SPADA: A Toolbox of Designing Soft Pneumatic Actuators for Shape Matching Based on Surrogate Modeling

**DOI:** 10.1089/rorep.2023.0029

**Published:** 2024-01-18

**Authors:** Yao Yao, Liang He, Perla Maiolino

**Affiliations:** ^1^ Oxford Robotics Institute, Department of Engineering Science, University of Oxford, Oxford, United Kingdom.; ^2^ Institute of Biomedical Engineering, Department of Engineering Science, University of Oxford, Oxford, United Kingdom.; ^3^ Department of Mechanical, Energy, Management and Transport Engineering—DIME, University of Genoa, Genoa, Italy.

**Keywords:** soft actuators, modeling, optimization, toolbox

## Abstract

Soft pneumatic actuators (SPAs) produce motions for soft robots with simple pressure input, however, they require to be appropriately designed to fit the target application. Available design methods employ kinematic models and optimization to estimate the actuator response and the optimal design parameters to achieve a target actuator's shape. Within SPAs, bellow SPAs excel in rapid prototyping and large deformation, yet their kinematic models often lack accuracy due to the geometry complexity and the material nonlinearity. Furthermore, existing shape-matching algorithms are not providing an end-to-end solution from the desired shape to the actuator. In addition, despite the availability of computational design pipelines, an accessible and user-friendly toolbox for direct application remains elusive. This article addresses these challenges, offering an end-to-end shape-matching design framework for bellow SPAs to streamline the design process, and the open-source toolbox SPADA (Soft Pneumatic Actuator Design frAmework) implementing the framework with a graphic user interface for easy access. It provides a kinematic model grounded on a modular design to improve accuracy, finite element method (FEM) simulations, and piecewise constant curvature (PCC) approximation. An artificial neural network-trained surrogate model, based on FEM simulation data, is trained for fast computation in optimization. A shape-matching algorithm, merging three-dimensional (3D) PCC segmentation and a surrogate model-based genetic algorithm, identifies optimal actuator design parameters for desired shapes. The toolbox, implementing the proposed design framework, has proven its end-to-end capability in designing actuators to precisely match two-dimensional shapes with root-mean-squared-errors of 4.16, 2.70, and 2.51 mm, and demonstrating its potential by designing a 3D deformable actuator.

## Introduction

Soft pneumatic actuators (SPAs) have a significant impact on the soft robotics field by enabling a variety of applications of soft robotics, such as grasping,^1,2^ locomotion,^3,4^ and manipulation.^5,6^ These versatile functionalities come from not only the inherent flexibility and adaptability of soft materials but also the capacity of SPAs to produce diverse two-dimensional (2D) and three-dimensional (3D) motion patterns.^7–9^ This is achieved by preprogramming the morphology of the actuator to achieve complex motions from a single pressure input, thus reducing the need of complex control.^10–13^

This trait has been widely employed in designing soft robots, especially those that draw inspiration from biological entities, as the desired actuator shape can be derived from creatures' movement. For instance, the locomotion pattern of inchworms has been replicated by designing actuators to recursively crawl,^4^ while the grasping profile of an elephant trunk has been emulated by actuators with helical configurations to achieve versatile and secure holds.^6^

Despite considerable advancements in discovering suitable soft actuator structures and materials to mimic biological motions, the design process is impeded by complexities tied to the dynamic, contact, and multimodal modeling of these actuators.^8,9^ Addressing these challenges remains difficult, given the limitation of current modeling and computational approaches in managing large geometric deformation and material nonlinearity.^14^ As a result, the research community's current primary focus lies in designing soft actuators for specific profiles or trajectories with kinematic models and optimization methods^15^ by establishing systematic methodologies and strategies to enhance efficiency.^12,15–17^

Fiber-reinforced SPAs, recognized for their versatile applications,^4,18,19^ leverage fiber arrangements for varied 2D and 3D motion.^10^ Connolly et al.^16^ presented an analytical model for these SPAs, comprising distinct modules for bending, twisting, elongating, and expanding. They took a predefined kinematic trajectory for each module and applied optimization to identify optimal actuator design parameters for matching shapes along the trajectory.

Singh and Krishnan,^17^ concentrating on bending modules, proposed a method that segments 2D curves into piecewise constant curvature (PCC) sections, with design parameters derived from their analytical model. However, the complex manufacturing of fiber-reinforced SPAs, especially the fiber routing process, faces challenges such as extended production time, inconsistency issues, and design limitations.

PneuNets actuators, formed of interconnected pleated channels within an elastomer, deform when pressurized.^20^ Their behavior is defined by altering channel geometry or material distribution.^11^ Advances in 3D printing allow these actuators to be crafted either *via* 3D-printed molds and silicon casting^15^ or direct 3D printing with flexible materials.^21^ Following Connolly et al.'s strategy,^16^ Jiang et al.^15^ modeled PneuNets, which include bending, twisting, and helical modules. Their design process starts with manual 3D curve segmentation, followed by their analytical model and optimization to determine the actuator design for the target shape.

Transitioning to bellow SPAs, replacing PneuNets' sharp channels with bellow-shaped convolutions, leads to an unfolding structure under pressure.^22,23^ This feature enables transferring material strain into structural deformation, making bellowSPAs particularly suitable for 3D printing materials such as Agilus30™ due to their lower elongation-at-break compared with silicon elastomers,^24^ ensuring precision in production. Kan et al.^12^ proposed an analytical model for modularized bellow SPA designs, using interconnected channels to achieve varied deformation curves. They employed a sampling-based optimization to design channels for a desired end-tip trajectory.

Analytical models, although widely used for forward kinematics, often struggle to accurately predict substantial deformations due to their reliance on geometric simplifications coupled with material nonlinearity. The finite element method (FEM) is known for its accurate predictions^25^ but is computationally expensive, making it unsuited for repeated optimization tasks.^26,27^ Recent advances use machine learning, particularly supervised artificial neural networks (ANNs) trained with FEM data, to create efficient surrogate models for bellow SPAs.^28^ However, these models have covered limited actuator design spaces.^29^

Furthermore, current shape-matching optimization methods for SPAs are limited by the need for human intervention in segmenting 3D shapes and in converting optimal parameters into a manufacturable design. This results in a lack of an end-to-end solution for seamlessly connecting the desired shape to a ready-to-print actuator design file. Furthermore, the inherent complexities of implementing design frameworks emphasize the need for an open-source user-friendly design toolbox to enhance accessibility and efficiency.^30^ Presently, the available toolboxes for bellow SPAs exhibit some limitations, either focusing solely on simulation or covering a limited design space.^31–33^ In contrast, some permit optimization but lack a specific focus on shape matching.^33,34^

**FIG. 1.**
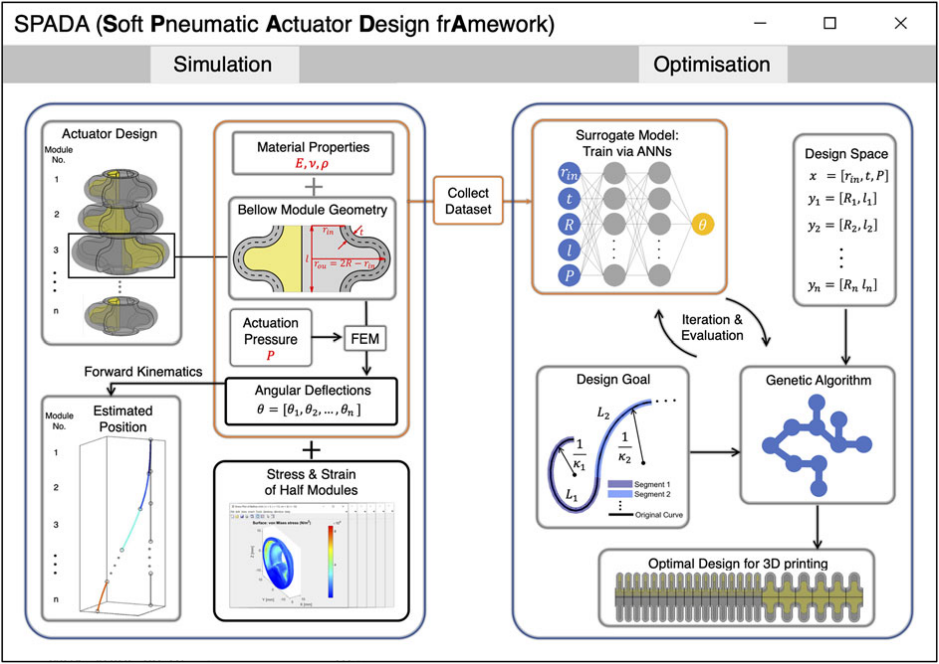
An overview of the bellow SPAs design toolbox, consisting of two main components: the simulation and the optimization components. The simulation component (left) facilitates the design of bellow SPAs by allowing modification of material properties, such as Young's modulus, Poisson's ratio, and density, adjusting the modular geometric parameters, and customizing module assemblies. It utilizes the FEM to simulate each module's angular deflection, mechanical stress, and strain based on the defined actuation pressure and predicts the actuator's configuration through forward kinematics. It also collects a data set of the FEM model. The optimization component (right) uses an ANN to train on the data set to provide a surrogate model for optimization. It takes an input of a target shape and divides it into constant curvature segments with length and curvature parameters using a 3D piecewise constant curvature segmentation. Then, a genetic algorithm is employed to optimize the parameters in the design space of a bellow SPA. Finally, a CAD file of the bellow SPA based on the optimal design parameter for matching the target shape can be generated. 3D, three-dimensional; ANN, artificial neural network; FEM, finite element method; SPA, soft pneumatic actuator.

Therefore, to address the above challenges, this article provides an end-to-end shape-matching design framework for bellow SPAs, and an open-source toolbox named SPADA (Soft Pneumatic Actuator Design frAmework), which implements this framework through a user-friendly graphic user interface (GUI). Figure 1 shows the schematic of the functions included in this design toolbox, consisting of simulation and optimization functionalities. The simulation component permits modular design customization, defining material properties, and predicting actuator kinematics using FEM combined with PCC approximation.

It also allows for collecting an FEM data set based on varied geometric parameters and actuation pressures. The optimization component can generate a surrogate model, trained on FEM or self-characterized data, to expedite computations. It segments input 2D/3D curves using PCC segmentation and uses the surrogate model to optimize the bellow SPA's design parameters for shape matching.

The rest of this article is organized as follows: Design and Methods section details the shape-matching design method, covering the bellow SPA's kinematics, FEM simulation, surrogate modeling, and the shape-matching optimization for determining design parameters. The Toolbox Implementation section introduces the SPADA toolbox, implementing the discussed framework with a user-friendly GUI for kinematic analysis and shape-matching optimization. Results section applies the toolbox for precise 2D shape-matching designs, also exploring 3D actuator design potential.

## Design and Methods

This section describes the methodologies employed for designing, analyzing, and optimizing bellow SPAs for shape matching. We present a modular design, describe its kinematics, discuss FEM simulations and ANN training, and introduce a shape-matching optimization algorithm for desired actuator shapes.

### Bellow SPAs design and kinematics

**FIG. 2.**
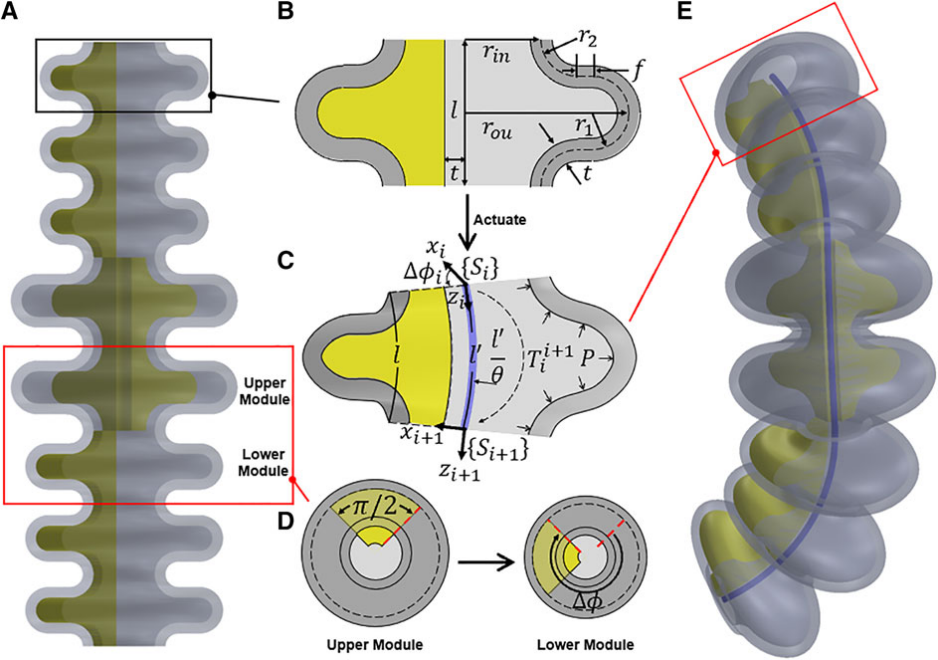
The design and kinematics of the bellow SPAs. (**A**) A bellow SPA consists of modules. (**B**) The parameterized 2D geometry of the module. (**C**) The deformed module and its kinematics model represented by an arc with length and curvature. (**D**) Rotation angle between adjacent modules. (**E**) The deformed bellow SPA upon pressurization with an inner line representing its deformed shape. 2D, two dimensional.

Modularity has demonstrated its effectiveness in exploring large design space of SPAs for achieving desired behaviors (Figure 2).^10–13^ This approach has been uniformly adopted in prior design methodologies for shape matching, owing to its capability to accelerate kinematics prediction and optimization.

As shown in Figure 2, a bellow SPA (Fig. 2A) can be built by stacking modules, whose deformation is determined by the deformation of each module along with the rotation angle between two adjacent modules. Each module consists of a U-shaped bellow ring and a $\frac{\pi }{2}$ fan-shaped constraint (Fig. 2B), parameterized by the geometric parameters given in Table 1.

**Table 1.** Geometric Parameters of the Bellow Soft Pneumatic Actuator Module

Parameters	Symbols/equations	Default values (mm)
Module length	*l*	10
Inner radius	$r_{in}$	5
Average radius	*R*	8
Outer radius	$r_{ou}=2R-r_{in}$	11
Upper radius	$r_{1}=l∕4$	2.5
Lower radius	$r_{2}=l∕4$	2.5
Wall thickness	*t*	1.5
Flank length	$f=r_{ou}-r_{in}-r_{1}-r_{2}$	1

Essentially, the geometry of the bellow SPA module is governed by four parameters $r_{in}$, *t*, *R*, and *l*, bounded by their non-negativity:

(1) $$\left\{\begin{array}{c} l>2t\\ R-r_{in}\geq \frac{l}{4} \end{array}\right..$$

The constraint restricts the expansion of the module, allowing it to unfold only in opposite directions during pressurization, resulting in a bending deformation of angle θ under the applied pressure *P*. Assuming the deformed module remains its original length along an axis on the side of constraint, offset by a distance of $r_{in}+\frac{t}{2}$ from the central axis, and the deformation follows the constant curvature (CC) approximation, it can be represented by an associate arc (indicated by a line) with the length l′ and curvature $\frac{\theta }{l\prime }$ (Fig. 2C), where $l\prime =\theta \left(\frac{l}{\theta }+r_{in}+\frac{t}{2}\right).$

As shown in Figure 2D, by having a clockwise rotation angle Δϕ between two adjacent modules (from upper to lower), the designed actuator can achieve spatial deformation, where the inner line indicates its deformed shape (Fig. 2E). To further simplify the design, all stacking modules share the same inner radius $r_{in}$, wall thickness *t*, actuation pressure *P*, and the manufacturing material. To this end, the kinematics model of bellow SPAs can be derived using the transformation matrix $T_{i}^{i+1}$mapping from the arc base frame $\left\{S_{i}\right\}$ to the tip frame $\left\{S_{i+1}\right\}$ of the *i*-th module, where

(2) $$T_{i}^{i+1}=\left[\begin{array}{cccc} c\Delta \phi_{i}c\theta_{i} & -s\Delta \phi_{i} & c\Delta \phi_{i}s\theta_{i} & \frac{l_{i}^{\prime }}{\theta_{i}}c\Delta \phi_{i}\left(1-c\theta_{i}\right)\\ s\Delta \phi_{i}c\theta_{i} & c\Delta \phi_{i} & s\Delta \phi_{i}s\theta_{i} & \frac{l_{i}^{\prime }}{\theta_{i}}s\Delta \phi_{i}\left(1-c\theta_{i}\right)\\ -s\theta_{i} & 0 & c\theta_{i} & \frac{l_{i}^{\prime }}{\theta_{i}}s\Delta \phi_{i}\\ 0 & 0 & 0 & 1 \end{array}\right],$$

in which *c* and *s* represent cos and sin.

### Surrogate model based on the FEM data set and ANN training

**FIG. 3.**
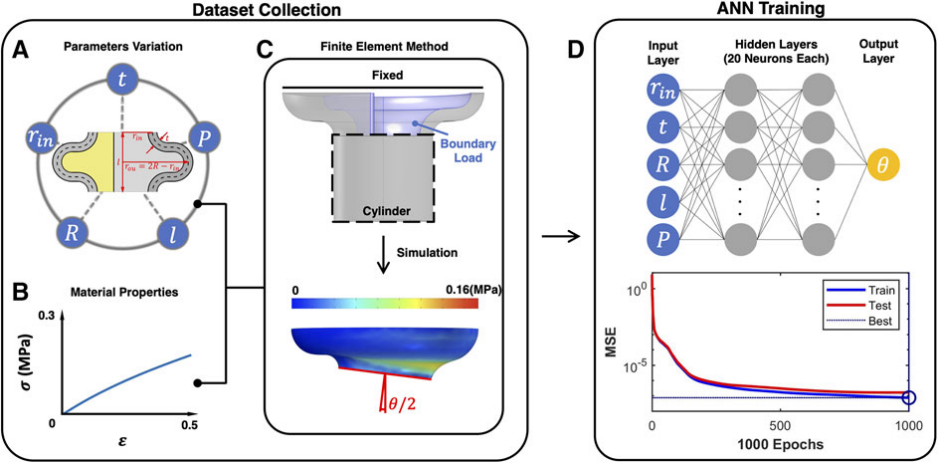
The process of generating the surrogate model. (**A**) The design parameters to be varied in data set collection. (**B**) The elastic properties of the material. (**C**) Boundary conditions of the half bellow module in the FEM simulation from the cross section perspective (top), and the deformed stress plot after simulation (bottom). (**D**) The ANN used to train the surrogate model based on the data set (top), and the MSE of both training and test data in relation to the change of epochs (bottom). MSE, mean squared error.

The above kinematics model for bellow SPAs maps arc parameters to actuator position but does not define the transformation from design space to arc parameters (Figure 3). Hence, a model connecting the design parameters (geometry and material properties) of a module to its deformation is needed. Figure 3 shows the surrogate modeling process using an FEM model for data set generation, subsequently training an ANN to expedite computation for optimization.

#### FEM simulation and data set collection

To generate a viable surrogate model of the bellow SPA, a data set should be obtained by sampling its feasible feature space. Four attribute geometry parameters and the actuation pressure are chosen as input to obtain a data set (Fig. 3A). The ranges and intervals of the selected input are set as given in Table 2.

**Table 2.** Range and Interval of Finite Element Method Simulation Input Data for Data Set Collection

Parameters	Minimum	Maximum	Interval
$r_{in}\left(mm\right)$	2	10	2
t(mm)	$r_{in}∕4$	$r_{in}∕3$	$r_{in}∕12$
R(mm)	$r_{in}+t$	$2r_{in}$	$\left(r_{in}-t\right)∕4$
l(mm)	4t	$4\left(R-r_{in}\right)$	$R-r_{in}-t$
P(kPa)	0	10	0.5

Considering the limitation of the material customizability, the data set is material specified. Therefore, a new set of collection is required for a new material. The incompressible Neo-Hookean hyperelastic material model is applied to the material (Fig. 3B):

(3) $$W=C_{1}\left(I_{1}-3\right)=\frac{\mu }{2}\left(I_{1}-3\right),$$

where $\mu =\frac{E}{2\left(1+\nu \right)}$ is the shear modulus, *I*_1_ is the first invariant of the right Cauchy–Green deformation tensor, *E* is Young's modulus, and ν is Poisson's ratio. Agilus30 is used as the default material as it is a commonly used material for designing soft robots.^35^ Material properties are obtained from a characterization work^36^ and an official datasheet.^24^

A MATLAB^®^ script is written to automatically create FEM simulations in COMSOL MultiPhysics^®^ based on the input and extract simulation results as output. Thanks to the symmetry of the bellow geometry, the FEM simulation is performed on the half bellow module as a stationary study of the 3D solid mechanics model (top of Fig. 3C). The top end is fixed, and the other is connected to a cylinder to smooth the element deformation. A uniformly distributed load is applied to the inner surface.

From the nonlinear simulation, deformation of the half bellow module and its von Mises stress distribution can be obtained (bottom of Fig. 3C). By using the displacement of a group of points at the end of the cylinder, the angular deflection θ of the bellow module can be calculated (see details of the FEM and material model in Section A in Supplementary Data).

#### Surrogate model trained *via* an ANN

To generate the surrogate model, a feedforward ANN (top of Fig. 3D) was constructed with an input layer of 5 neurons for the input data (consisting of 4 geometric parameters and the actuation pressure based on Table 2), 2 hidden layers of 20 neurons each and an output layer of 1 neuron for the corresponding output data (angular deflection). The mean squared error (MSE) served as the performance function. The Bayesian regularization backpropagation is used as the training function.

The precollected FEM simulation data set is randomly divided into training and test sets (80% and 20%, respectively) and trained until 1000 epochs have been reached. After training, the MSE reaches <$10^{-6}$ for both training and test data (bottom of Fig. 3D). This pretrained network will be then used as the surrogate model in the form of $\theta =f\left(r_{in},t,R,l,P\right)$ to quickly predict the angular deflection of the bellow SPA module for a given set of design parameters, allowing for efficient design exploration and optimization.

### Shape matching optimization

**FIG. 4.**
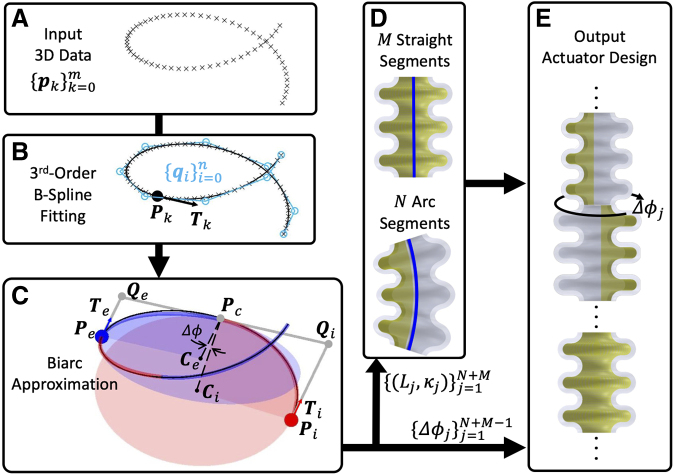
The shape-matching optimization process, explained in Shape Matching Optimization section in detail. (**A–C**) Shows the piecewise constant curvature segmentation algorithm, in which the input data ${\left\{p_{k}\right\}}_{k=0}^{m}$ are first fitted by a third-order B-spline curve controlled by light blue control points ${\left\{q_{i}\right\}}_{i=0}^{n}$, with projected point ***P***_*k*_ and its tangent vector ***T***_*k*_; then discretised into constant curvature segments using the biarc approximation by interpolating between two end points ***P***_*i*_, ***P***_*e*_ and their tangent vectors ***T***_*i*_, ***T***_*e*_, based on the control points ***Q***_*i*_, ***Q***_*e*_ and the connection point ***P***_*c*_; the rotation angle Δϕ between two segments is $∠C_{i}P_{c}C_{e}$. (**D**) Shows that for each CC segment represented by shape parameters $\left(L_{j},\kappa_{j}\right)$, a module will be designed to match its shape by stacking one or more identical modules. (**E**) Shows the output actuator is designed by stacking the previous modules in order with rotation angles $\Delta \phi_{j}$ between segments.

Given a curve represented by a set of 3D ordered points ${\left\{p_{k}\right\}}_{k=0}^{m}$, we aim to design a bellow SPA to deform from an unactuated straight line to a desired shape upon pressurization (Figure 4). Figure 4 outlines our shape-matching process. Initially, a 3D PCC segmentation algorithm discretizes the curve into CC segments (Fig. 4A–C). Next, the actuator's design involves determining the optimal module parameters to match each CC segment (Fig. 4D, E). Notably, “segment” pertains to the desired shape's divided part, whereas “module” refers to the bellow SPA's building block. Each segment's shape is approximated using one or multiple identical modules.

PCC requires the curve to follow *G*^1^ continuity, meaning two adjacent CC segments share an endpoint with aligned tangent vectors. The biarc method, frequently used for this,^37^ requires two endpoints ***P****_i_*, ***P****_e_* and their tangents ***T****_i_*, ***T****_e_* (Fig. 4C). The challenge is determining the connection point ***P****_c_* between two CC segments:

(4) $$P_{c}=\frac{d_{2}\left(P_{i}+d_{1}T_{i}\right)+d_{1}\left(P_{e}-d_{2}T_{e}\right)}{d_{1}+d_{2}},$$

where $d_{1}=\left|Q_{i}-P_{i}\right|=\left|Q_{i}-P_{c}\right|$ and $d_{2}=\left|Q_{e}-P_{e}\right|=\left|Q_{e}-P_{c}\right|$, with ***Q****_i_* and ***Q****_e_* as biarc control points. The relationship $\left|Q_{e}-Q_{i}\right|=d_{1}+d_{2}$ allows solving the biarc with $d_{1}=d_{2}$. Furthermore, the rotation angle Δϕ between two CC segments is determined by the angle between $C_{i}P_{c}$ and $C_{e}P_{c}$, where ***C****_i_* and ***C****_e_* are the respective circle centers.

The biarc approximation samples data points, starting from the initial point, seeking the longest biarc that approximates data within a given tolerance. This process is iteratively repeated until all data are approximated.^38^ Tangent vectors are derived using a third-order B-spline curve fitted to the original 3D data (Fig. 4B), enabling the calculation of projection points and their tangent vectors. This B-spline curve is defined by n+1 control points ${\left\{q_{i}\right\}}_{i=0}^{n}$:


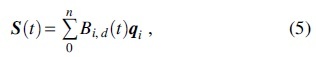


where d=3−1 is the degree of curve, 3 is the order of curve, *t* is a sequence of nondecreasing uniform knots such that $t_{i}=\frac{i-d}{n+1-d}$ for d+1≤i≤n, and there is d+1 number of 0 and 1 at the beginning and end of the knot sequence, respectively. *B* is the basis function that is defined recursively:

(6) $$B_{i,0}\left(t\right)=\left\{\begin{array}{c} 1,t_{i}\leq t\leq t_{i+1}\\ 0,otherwise \end{array}\right..$$

(7) $$B_{i,j}\left(t\right)=\frac{t-t_{i}}{t_{i+j}-t_{i}}B_{i,j-1}\left(t\right)+\frac{t_{i+j+1}-t}{t_{i+j+1}-t_{i+1}}B_{i+1,j-1}\left(t\right).$$

Assuming that the sample sequence of the input data points is $t_{k}=\frac{k}{m}$, matrix (insert-EQ) composed of the control points of the B-spline that fits the data can be obtained using the least-squares fitting,

(8) $$\hat{q}=\left({\left(A^{T}A\right)}^{-1}A^{T}\right)\hat{p},$$

where $A=\left[B_{i,d}\left(t_{k}\right)\right]$ and $\hat{p}$ is the matrix composed of all data points, and the number of control points n+1 can be determined by minimizing the error from input data points to the B-spline curve.^39,40^ Then, all data points are projected to the B-spline curve to get the projected points ${\left\{P_{k}\right\}}_{k=0}^{m}$ and their tangent vectors ${\left\{T_{k}\right\}}_{k=0}^{m}$.

A number of CC segments are obtained after the PCC segmentation (Fig. 4D). If a segment curvature is <$10^{-3}$, it is deemed straight. These segments are designed by stacking modules filled with internal constraints. For arc segments, a genetic algorithm determines the bellow SPA module design parameters for matching shapes by stacking identical modules. The selection of the genetic algorithm is informed by our previous study,^41^ thanks to its compatibility with surrogate modeling and its capacity to effectively handle a large number of design variables.

The objective is to minimize the difference in arc parameters (arc length and curvature) between the deformed segment shape (composed of designed modules and predicted by a surrogate model) and the desired segment shape (see details in Section B in Supplementary Data). The final actuator is constructed by orderly stacking these modules, considering rotation angles between segments (Fig. 4E).

## The Toolbox Implementation

**FIG. 5.**
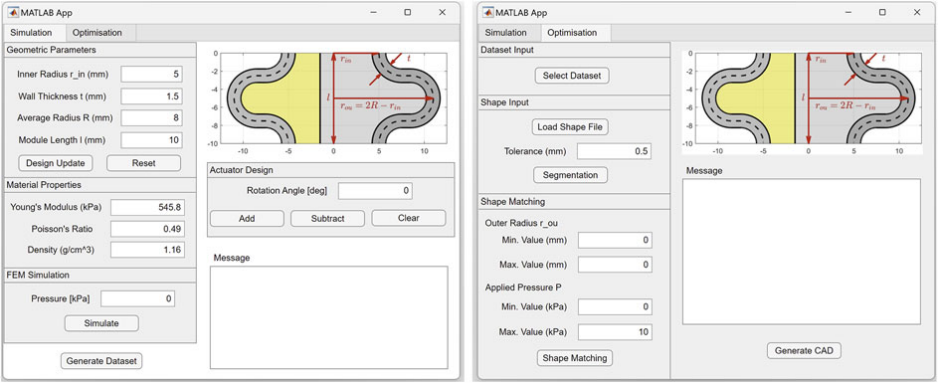
The graphic user interfaces of the toolbox SPADA, including the simulation (left) and optimization (right) components. SPADA, Soft Pneumatic Actuator Design framework.

To amplify the efficiency and user accessibility of our design framework, we have developed SPADA—an open-source design toolbox with a user-friendly GUI (Fig. 5)—built on MATLAB and COMSOL MultiPhysics. Compared with our previously published bellow SPA design toolbox—designed solely for simulating bending actuator behaviors and taking ∼74 minutes per simulation^31^—SPADA stands out by facilitating simulations for diverse deformations in just a few minutes and offering efficient end-to-end shape-matching optimization. Here, “end-to-end” refers to the process from the desired shape to the stereolithography files of the designed actuator for 3D printing.

The simulation component allows for the design of bellow SPAs by adjusting the modular geometric parameters, customizing assemblies, and modifying material properties. It utilizes background FEM simulation to analyze the behavior of modules and predict the actuator's configuration through forward kinematics. In addition, it can generate material-specified data sets.

The optimization component can train a selected data set into an ANN to serve as a surrogate model. It takes as input a file containing the desired shape of the actuator, represented as a series of ordered 3D points. The 3D PCC segmentation algorithm segments the desired shape into CC segments, and an optimization algorithm based on a genetic algorithm and the surrogate model is used to find the optimal actuator design parameters that approximate the desired shape. It can also generate computer-aided design files of the designed actuator, ready for direct 3D printing.

See Section C in Supplementary Data for detailed instructions on how to use SPADA. The source code is available on a GitHub repository,^42^ and a demonstration of the toolbox is provided in the Supplementary Video S1.

## Results

In this section, SPADA was used to design three actuators that accurately match the predefined 2D shapes with root-mean-squared-errors (RMSEs) of 4.16, 2.70, and 2.51 mm, respectively, hence validating the accuracy of the kinematics model, illustrating the efficacy of the shape-matching algorithm, and demonstrating its ability in achieving end to end from desired shapes to designed actuators for direct 3D printing. Furthermore, we harnessed the toolbox's potential in the design of 3D deformable actuators, specifically by designing an actuator according to an elephant trunk-inspired helical shape.

### 2D shape matching: letter writing

**FIG. 6.**
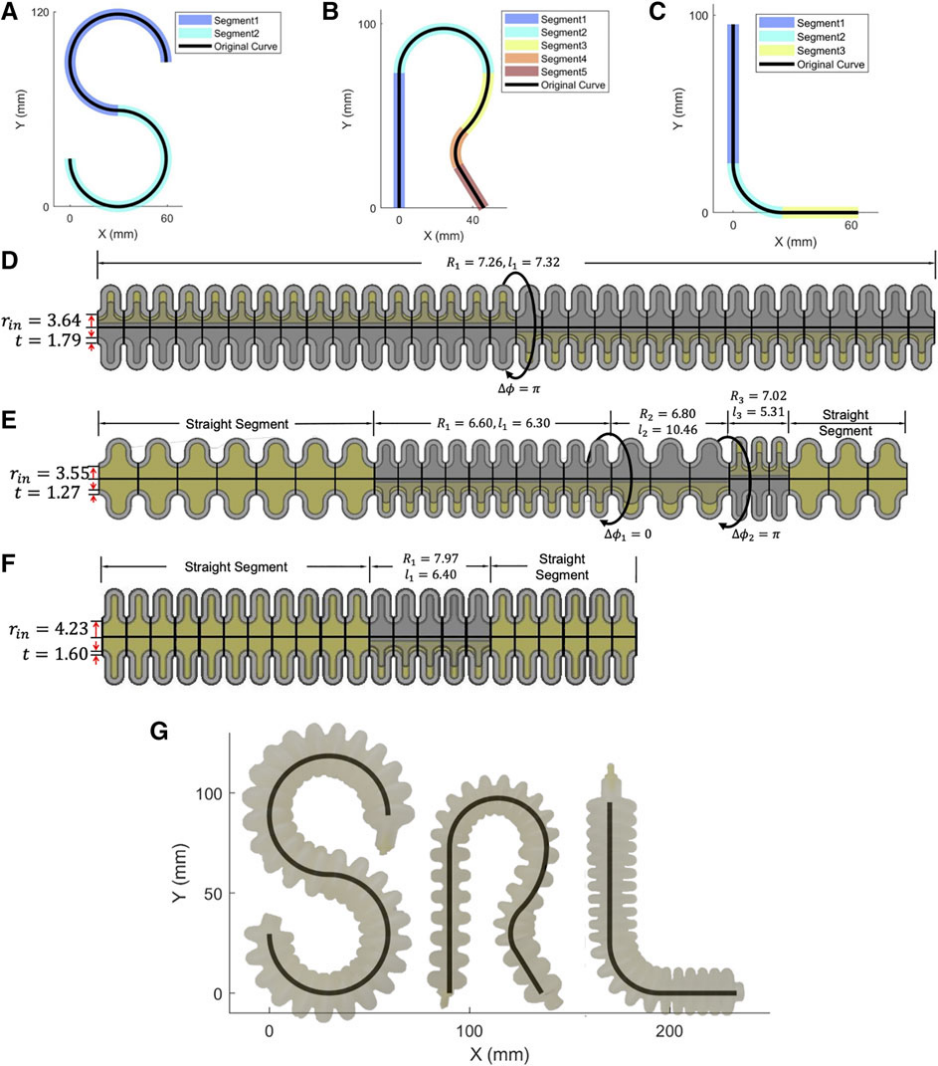
The 2D shape-matching case: the input shapes of letters “SRL” are segmented in (**A–C**). (**D–F**) Are the actuator schematics that match the input shapes and designed by optimal parameters. (**G**) Shows the deformed prototypes compared with the input shapes.

In the 2D case of validating the shape-matching algorithm along with the kinematics model, and also demonstrating the ability of end to end from the desired shapes to 3D-printed actuators, SPADA was used to design three actuators that match the shape of the letters “S,” “R,” and “L,” which is the acronym of Soft Robotics Lab (Figure 6).

The shape for each letter is represented by a series of ordered 3D coordinates, imported into SPADA as a “.mat” file. The segmentation results for each letter are highlighted in Figure 6A–C. The letter “S” is segmented into two arc segments with the same length and curvature. The letter “R” is segmented into two straight segments and three different arc segments. The letter “L” is segmented into two straight segments and one arc segment. All the lower bound values of the outer radius were set to be 9 mm for three actuators optimization for ease of manufacturing.

After optimization, the optimal design parameters are labeled on the schematics of actuators designed by those parameters in Figure 6D and E. The surrogate model used was provided with the toolbox, which was trained by a provided FEM data set of the default material Agilus30 (see information of the FEM and material parameters in Section A in Supplementary Data). The prototypes were 3D printed on a Stratasys J735™ using the CAD files generated by SPADA.

The shape of the actuated prototypes was compared with the initial desired shape shown in Figure 6G by overlapping them in the *x*–*y* plane. The respective RMSEs between the desired shapes and actuators' real shapes are 4.16, 2.70, and 2.51 mm, validating and demonstrating our shape-matching algorithm (refer to Section D.I in Supplementary Data for details of the experiments, and see the prototypes taking the defined shapes in the Supplementary Video S1 available on the project's GitHub page^42^).

### 3D shape matching: elephant trunk-inspired shape

**FIG. 7.**
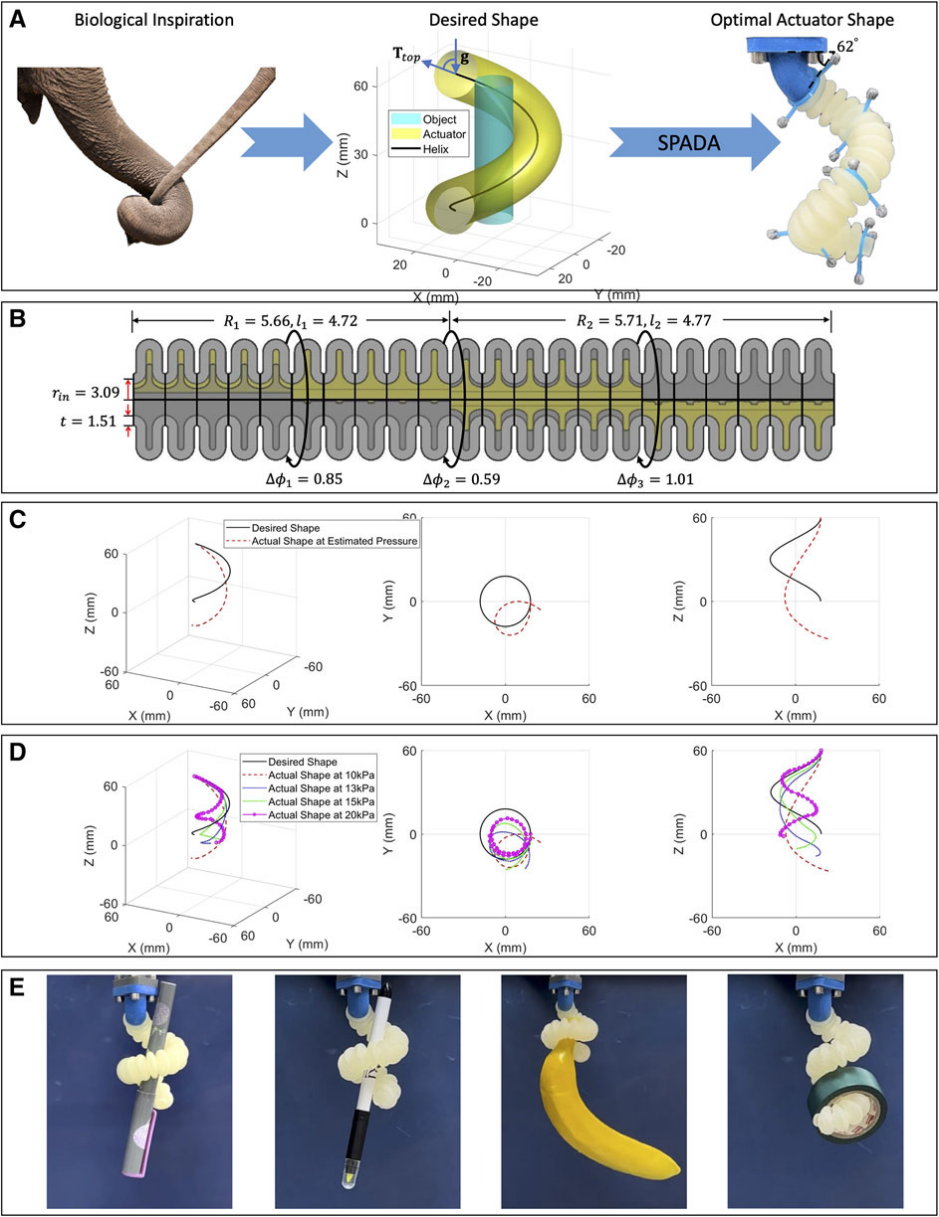
The 3D shape-matching case: (**A**) depicts the process of inspiration drawing from an elephant trunk (edited image © Shutterstock, accessed on August 1, 2023), acquiring the helical shape, and subsequent design of the actuator using our toolbox SPADA. (**B**) Showcases a schematic of the actuator with optimal design parameters. (**C**) Provides a comparison between the desired helical shape and the actual shape of the actuator under the pressure estimated by the toolbox. (**D**) Compares the desired shape and the actual shapes of the actuator under escalated pressure. (**E**) Demonstrates the actuator's versatility in handling and grasping various objects.

The previous 2D case of shape matching validates the efficacy of our end-to-end design framework, given its independence from gravitational effects (Figure 7). Yet, in the 3D scenario, gravity is unavoidable. Furthermore, gravity acts unidirectionally while the deformation of actuators significantly varies in space, making it exceedingly challenging to incorporate gravity's impact into the modeling process, particularly in modular approaches. This complexity explains why nearly all shape-matching design frameworks sidestep considering gravitational effects.^12,16,17^ Although Jiang et al.^15^ did incorporate gravity into their analytical model, they were only able to roughly approximate its influence for two types of segments and did not evaluate it in the real world.

We recognize that shape matching, on its own, cannot offer a comprehensive solution for tasks involving gravity and contact. Yet, our design framework and toolbox still provide valuable insights for actuator design, even without considering these factors, thanks to the adaptability of soft actuators. The design of actuators to assume a helical shape, emulating the versatile and secure grasping behavior of an elephant's trunk, is a prime example among the research community.^6,21,43^

Hence, both to demonstrate the potential of the toolbox for employing 3D shape matching in guiding actuator design and to quantitatively assess gravity's impact on the 3D shape-matching scenario, we delve into an end-to-end example of designing a helical actuator, inspired by the structure of an elephant's trunk, for the purpose of grasping irregular objects, as shown in Figure 7.

A right-handed helix, with x,y,z coordinates represented by the mathematical expression $\left(a\cos t,a\sin t,ht\right)$, where *t* is evenly spaced between 0 and 2π, is utilized to embody the helical configuration inspired by the grasping movement of an elephant's trunk. By presuming that the object targeted for grasping fits within a cylindrical dimension of 16mm diameter and 120mm height, and further constraining the actuator's outer radius to be ∼10 mm, the helix parameters a=18mm and h=60mmcan be derived for a power grasp.

The helical shape, consisting of a sequential of ordered 3D points, is imported into SPADA. An actuator is then designed to match this shape using the toolbox framework while disregarding gravitational effects. Figure 7B showcases the design parameters of the actuator, and the toolbox estimates the actuation pressure to be 9.85kPa (which is approximated to 10kPa in experiments due to the hardware limitations). To assess the discrepancy between the desired shape and the actual shape of the pressurized actuator under the effect of gravity, we executed a series of experiments. The top end of the actuator is fixed to a connector with an angle of $62^{\circ }$ (Fig. 7A). This angle corresponds to the divergence between the tangent vector at the helix's top end and the gravity axis.

We first pressurized the actuator to 10kPa, a value estimated by the toolbox as optimal for achieving the helical shape. Figure 7C illustrates the deviation between the desired helical shape and the actual configuration of the actuator at this pressure. The RMSE is calculated to be 25.64mm.

Subsequently, we escalated the actuation pressure to13, 15, and 20kPa. Figure 7D compares the desired shape and the actuator's actual shapes under these increased pressure values. The calculated RMSEs for these pressures were19.08, 18.69, and 23.77mm, respectively. Moreover, as observed from the *XY* and *XZ* perspectives in Figure 7C and D, it is evident that as pressure increases, the central point of the actual shape (*XY* view) approaches that of the targeted shape.

Concurrently, the number of convolutions in the actual shape (*XZ* view) increases, whereas the height of the actual shape along the *z*-axis decreases. This phenomenon can be attributed to the escalating pressure that not only counteracts gravitational effects but also enhances the curvature perpendicular to the direction of gravity. This, in turn, induces an increase in the number of helical turns.

However, despite the error caused by gravity, it is still possible to exploit behaviors arising from the design and interaction with the object to achieve a successful grasp. The grasping experiments were performed to explore the potential of the designed actuator for handling various objects, as shown in Figure 7E. Refer to Section D.II in Supplementary Data for details of the grasping experiments and watch the prototype grasping objects in the Supplementary Video S1.

## Conclusion

In this study, an end-to-end design framework for the shape matching of bellow SPAs is proposed and implemented as an open-source design toolbox, SPADA. A model leverages FEM-simulated module deformation and PCC approximation to predict the kinematics of actuators. Surrogate modeling involving ANN training on a FEM data set is applied to speed up computation. A 3D PCC segmentation algorithm approximates the desired curve by dividing it into CC segments.

An optimization algorithm, grounded on a genetic algorithm and a surrogate model, determines the optimal design parameters of the actuator to align with the shape of these segments. The toolbox, based on the proposed design framework, has proven its end-to-end capability in designing actuators to accurately match 2D shapes, while also demonstrating its potential to design deformable actuators in 3D space.

Overall, this design framework can be generalized to other soft actuators, such as tendon-driven soft actuators. Users can also specify more material properties for the actuator and use the toolbox FEM simulation function to create a data set for training. A GitHub repository has been created with the toolbox and locations for users to upload self-acquired data sets for codesign. In the future, the authors will maintain and update the toolbox with consideration of the gravity effect and environmental parameters (such as underwater conditions). Add-ons for protocols to design soft actuators for specific tasks will also be investigated, such as soft grippers, soft actuators for surgical operations, and soft crawlers for locomotion.

## Abbreviations Used

2D: two-dimensional
3D: three-dimensional
ANN: artificial neural network
CC: constant curvature
FEM: finite element method
MSE: mean squared error
PCC: piecewise constant curvature
RMSE: root-mean-squared-error
SPA: soft pneumatic actuator
SPADA: Soft Pneumatic Actuator Design frAmework
