# Supplementary material for: SPADA: A Toolbox of Designing Soft Pneumatic Actuators for Shape Matching Based on Surrogate Modeling
Source: Robot Rep. 2024 Jan 18;2(1):1–14. doi: 10.1089/rorep.2023.0029 (PMC11386149; doi:10.1089/rorep.2023.0029)
Supplement: Supplementary Data [file rorep.2023.0029_suppl_data.pdf]

SUPPLEMENTARY DATA

## *A. The FEM Simulation and Material Model*

The FEM simulation is performed as a stationary study of the 3D solid mechanics model in COMSOL MultiPhysics® 5.6a. The half bellow module is modelled using parameters in Table 1, with the incompressible Neo-Hookean hyperelastic material model in Eq.3, factoring in uniaxial deformation.

The rationale behind the material model selection is twofold:

(1) it is a simple material model that only requires a shear modulus, which can be approximated by Young's modulus and Poisson's ratio, making it easy for users to find parameters for their own materials;

(2) this model offers a satisfactory agreement for lower strain values, typically below 50% as reported[25]. Furthermore, based on our previous investigations[31], the mechanical strain for bellow-SPAs primarily remains under 50% for an actuation pressure range of 0-10kPa. Consequently, the neo-Hookean model has been deemed an appropriate choice.

Regarding material properties, we've set Poisson's ratio to 0.49, and the material density $\rho = 1.16\ \mathrm{g/cm^3}$ is obtained from the official data sheet[24]. Unable to conduct direct experimental characterization of the material, we derived Young's modulus by linear fitting the Ogden model data of Agilus30™, as presented in the work of Abayazid and Ghajari[36], within a strain range of 0-1. This range was chosen as the data exhibited a consistent slope up to this point, with a noticeable change in slope for strains exceeding 1. As we couldn't access the original experimental data from their work, our comparison between the neo-Hookean model and their Ogden model for the given strain range yielded a Root Mean Square Error (RMSE) of 20% as indicated in Fig.S1 below.

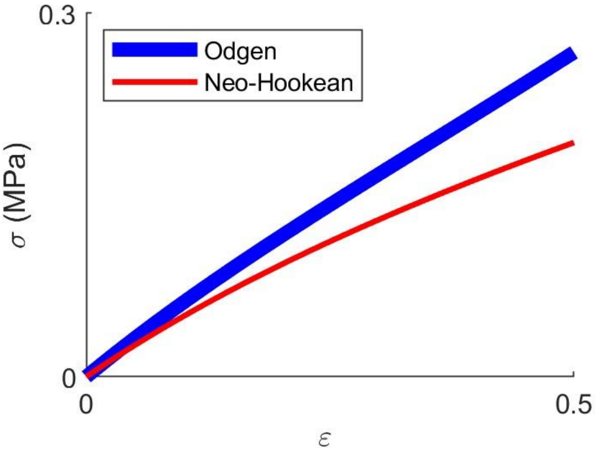


Fig S1. The comparison between the Ogden model data (from the work of Abayazid and Ghajari[36]) and the Neo-Hookean model (using the linearly fitted Young's Modulus) for Agilus30™ in the strain range of 0-0.5.

Furthermore, the accuracy of the kinematic shape-matching is affected by the accuracy of the material model. From the experimental results of the 2D shape-matching example, it is possible to notice that despite the discrepancy between the two material models, we achieved a very good match.

In the FEM model, the cylinder used to smooth its element deformation has a radius of $r_{in} + \frac{t}{2}$ and a length of $l$ connected to the smaller end of the half bellow using the same material model. For the simulation using Agilus30™ as material, the Young's Modulus 546 kPa is determined by linear fitting the Odgen Model with parameters from the work of Abayazid and Ghajari[36], the Poisson's ratio is set as 0.49, and material density is set as 1.16 g/cm$^3$. The pressure is modelled as the uniformly distributed load on the inner surface of the half bellow, ramped by intervals of 0.5 KPa. The mesh is set as the default element size of “Finer”. To allow for large deformation of elements, the geometric nonlinearity is included, with “Constant Newton” and “Anderson acceleration” set as the nonlinear simulation method.

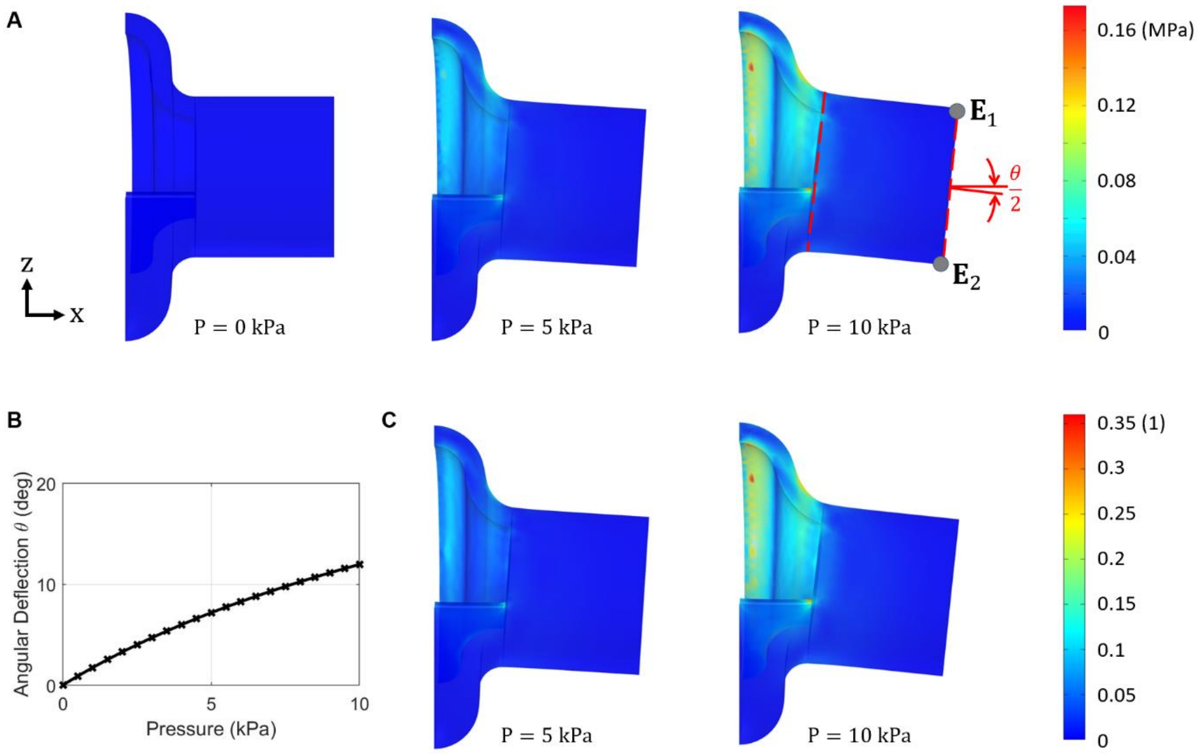


Fig.S2: The detailed FEM simulation results of the bellow SPA module with the default geometric parameters (Table 1) and the default material Agilus30™. A. The von Mises stress distribution of half FEM model changes with increased pressure. B. The angular deflection $\theta$ of the module predicted by the FEM model change with increased pressure. C. The first principal strain distribution of half FEM model changes with increased pressure.

Assuming the plane where the cylinder is connected to the bellow is parallel to the plane at the other end of the cylinder, the angular deflection of the half bellow is the angle between this plane and the plane at the fixed end of the half bellow. Therefore, the angle can be calculated by the displacement of the top point $E_1$ and the bottom point $E_2$ in the plane at the free end of the cylinder (as shown in Fig.S2(A). If the coordination of $E_1$, $E_2$ are $(x_1, y_1, z_1)$ and $(x_2, y_2, z_2)$, respectively. Then the angular deflection of the module can be calculated by

$$\theta = \cos^{-1}\frac{z_1 - z_2}{\sqrt{(z_1 - z_2)^2 + (x_1 - x_2)^2}} \quad \text{(S1)}$$

Additionally, the FEM simulated stress distribution, angular deflection and strain distribution of the bellow SPA model with default design parameters in relation to the change of pressure are shown in Fig.S2.

### *B. The Optimization Algorithm*

Numerous optimization algorithms are available, as detailed in the review[26]. Based on our prior investigation into optimization algorithms suitable for bellow-SPAs shape-matching design[41], we identified the genetic algorithm as a prime choice for this work. This choice was influenced by its compatibility with surrogate modeling and its efficiency in handling large design variable dimensions.

Despite being provided in our previous work, we revisit the optimization algorithm here by integrating a genetic algorithm, a surrogate model, and the symbols introduced in this study. Assuming there are in total $N$ unique arc segments with length and curvature pairs $[(L_1, \kappa_1), (L_2, \kappa_2), \dots, (L_N, \kappa_N)]$ different from each other, there will be $N$ different bellow SPA modules, each approximating one segment.

We wish to determine their shared parameters $\mathbf{x} = [r_{in}, t, P] \in \mathbb{R}_+^3$ and a set of respective geometric parameters $\mathbf{y} = [\mathbf{y}_1, \mathbf{y}_2, \dots, y_N] = [(R_1, l_1), (R_2, l_2), \dots, (R_N, l_N)] \in \mathbb{R}_+^{2N}$. The optimization objective is to minimize discrepancies in arc parameters (arc length and curvature) between the desired shape $\{(L_j, \kappa_j)\}_{j=1}^N$ and the deformed shape of the actuator designed by $\mathbf{x}, \mathbf{y}$, which is constructed from designed modules and predicted by a surrogate model. We have a total of $2N + 3$ optimization variables for one actuator, as shown in Fig.S3.

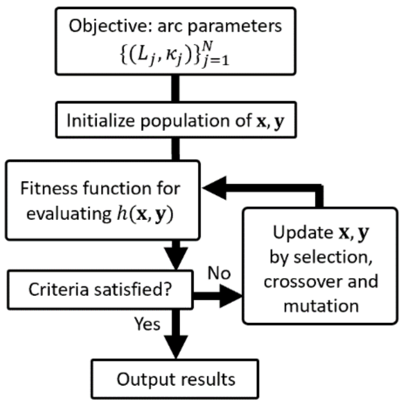


Fig.S3: The optimization algorithm of designing bellow SPAs for shape-matching.

The constraints are specified by the maximum actuation pressure $P_{\max}$, the maximum curvature of segments $\kappa_{\max}$, the segment length and the inherent design constraints of the bellow SPA module. The problem is formulated as follows:

$$\min h(\mathbf{x}, \mathbf{y}) := \frac{1}{N} \sum_{j=1}^{N} d_j(\mathbf{x}, \mathbf{y}_j) \tag{S2}$$

$$\text{s.t.} \begin{cases} 0 \leq P \leq P_{max} \\ 0 < r_{in} \leq \dfrac{1}{\kappa_{max}} \\ \dfrac{R_j}{4} \leq t \leq \dfrac{r_{in}}{2} \\ r_{in} + t \leq R_j \leq \min\left(\dfrac{1}{2\kappa_j} + \dfrac{r_{in}}{2}, 2r_{in}\right) \\ 2t < l_j < \min\left(L_j, 4\left(R_j - r_{in}\right)\right), \forall j \in [N] \end{cases} \tag{S3}$$

where

$$d_j\left(\mathbf{x}, \mathbf{y}_j\right) = w_1 \left|L_j - n_j l_j'\right| + w_2 \left|\kappa_j - \frac{\theta_j}{l_j'}\right| \tag{S4}$$

In which $l_j' = \theta_j \left(\frac{l_j}{\theta_j} + r_{in} + \frac{t}{2}\right)$ is module's deformed length, $n_j = \left\lceil \frac{L_j}{l_j'} \right\rceil$ is the number of identical modules in the $j$-th segment, and $w_1 = \frac{1}{L_j}$, $w_2 = \frac{1}{\kappa_j}$ are the weighting factors used to balance the difference of units. $\theta_j = f(r_{in}, t, P, R_j, l_j)$ is obtained from the surrogate model trained before.

Beyond the default settings of genetic algorithms in MATLAB®, we established stopping criteria for either reaching 500 iterations or the objective function dropping below 0.02. If a feasible point is found, the optimization outputs the shared parameters $[r_{in}, t, P]$ and the respective parameters $[R, l]$ for each segment.

### C. *SPADA User Guide*

#### I. *The Simulation Component*

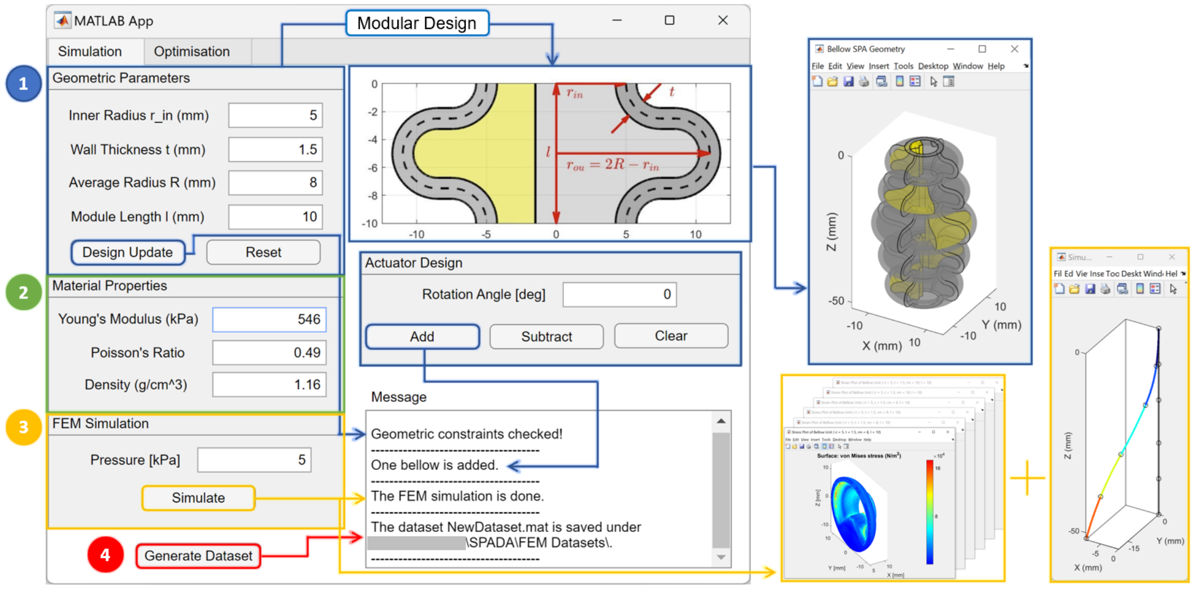


Fig.S4: The GUI and usage instructions of the simulation component in the toolbox (information for each step is highlighted in the same color as the step number icon)

The interface of the simulation component is shown in Fig.S4.

1) The "Geometric Parameters" section allows entering four geometric parameters of the bellow SPA module. The "Design Update" button checks geometric constraints and updates the 2D schematic based on the input, while the "Reset" button resets them all to the default values. The "Actuator Design" section constructs the actuator in a modular approach (an example is shown in a blue box on the right side). The "Add" button stacks modules based on the current geometric parameters and orientation angles; "Subtract" removes the last added module, while "clear" removes all.
2) The "Material Properties" section allows for defining the material of the actuator by its Young's modulus, Poisson's ratio and density.
3) The "FEM Simulation" section takes the input of the actuation pressure; the "Simulation" button launches COMSOL in the background and analyses the designed actuator using FEM. It simulates the behavior of each unique module and predicts the configuration of

the whole actuator with forward kinematics (examples are shown in the first and second yellow boxes on the right side, respectively).

4) The "Generate Dataset" button uses FEM to collect a dataset as explained in Sec.2.B.I based on the input material properties. A dataset for Agilus30™ is already collected and provided to users.

## II. *The Optimization Component*

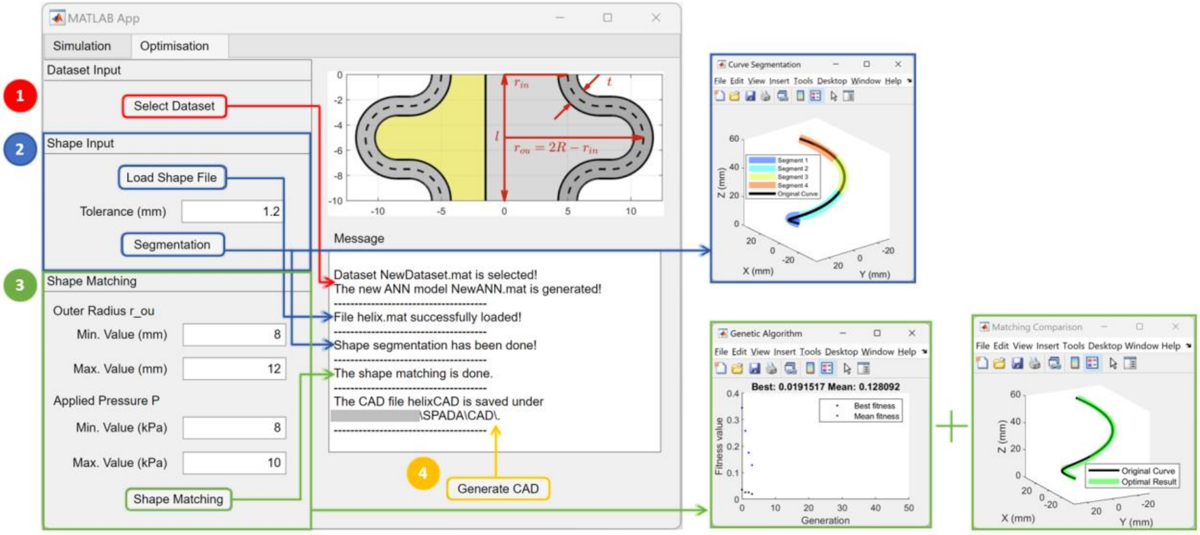


Fig.S5: The GUI and usage instructions of the optimization component in the toolbox (information for each step is highlighted in the same color as the step number icon)

The interface of the optimisation component is shown in Fig.S5.

1) In the "Dataset Input" section, the "Select Dataset" button trains a selected dataset into a surrogate model via an ANN, as described in Sec.2.B.II. The dataset can be obtained by executing step 4 in the toolbox's simulation component, with user-defined material properties. Alternatively, users can create their own datasets, ensuring matching the format of our provided dataset for Agilus30™, available on our toolbox's GitHub repository[42].

2) In the "Shape Input" section, the desired shape file consisting of 3D coordinates of sequential points can be input with the "Load Shape File" button. The

"Segmentation" button helps divide the 3D shape into straight and circular arc segments by the 3D PCC segmentation algorithm according to the input tolerance value.

3) The "Shape Matching" section uses a genetic algorithm to find and output the optimal design parameters of the actuator that matches the shape upon pressurization based on the input upper and lower bounds of the actuator's outer radius and the applied pressure.

4) If the algorithm can find a set of feasible design variables, the "Generate CAD" button can generate a .stl format file of the designed actuator for direct 3D printing.

## D. *Experiment Details*

### *I. The 2D Shape-Matching*

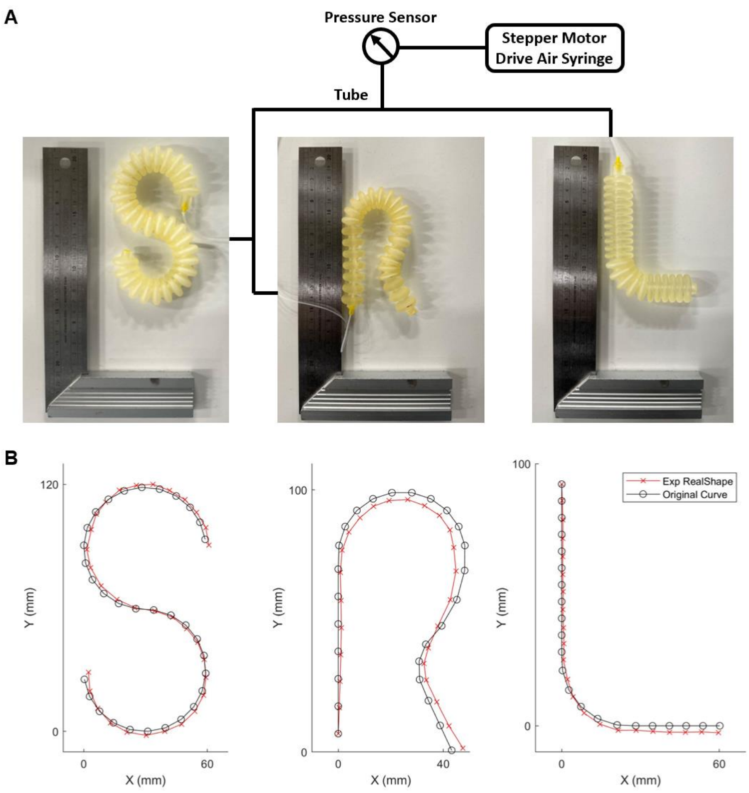


Fig.S6: A) The setup for the 2D shape-matching experiment (top). B) The comparison between the original letter shapes and actuators' real shapes obtained through experiments (bottom).

The setup of the experiment in Sec.4A is shown in Fig.S6. Three actuators “S”, “R”, “L” were driven with respective optimal pressures 8.11, 6.97 and 6.94kPa (which were approximated to 8, 7 and 7kPa in experiments due to the hardware limitations), determined along with their design parameters by the shape-matching design framework implemented on SPADA. A pneumatic system consisting of a 12V DC stepper motor driving a 60mL air syringe, a micro-controller (Arduino Uno), a pressure sensor (ADP51A11, pressure range 0-40 kPa, Panasonic, Japan) and a PI controller was used to control the input pressure. Lubricant was applied beneath the actuators to remove frictional effects. After taking photos from actuators’ upward direction, the MATLAB Image Processing Toolbox™ was used to identify their real shapes by getting the central pixel coordinates of each module and using the steel square as reference. The original letter curves were sampled with the same intervals to compare with the real shapes. The root-mean-squared-errors for three actuators are 4.16, 2.70, and 2.51mm, respectively.

### *II. The 3D Shape-Matching*

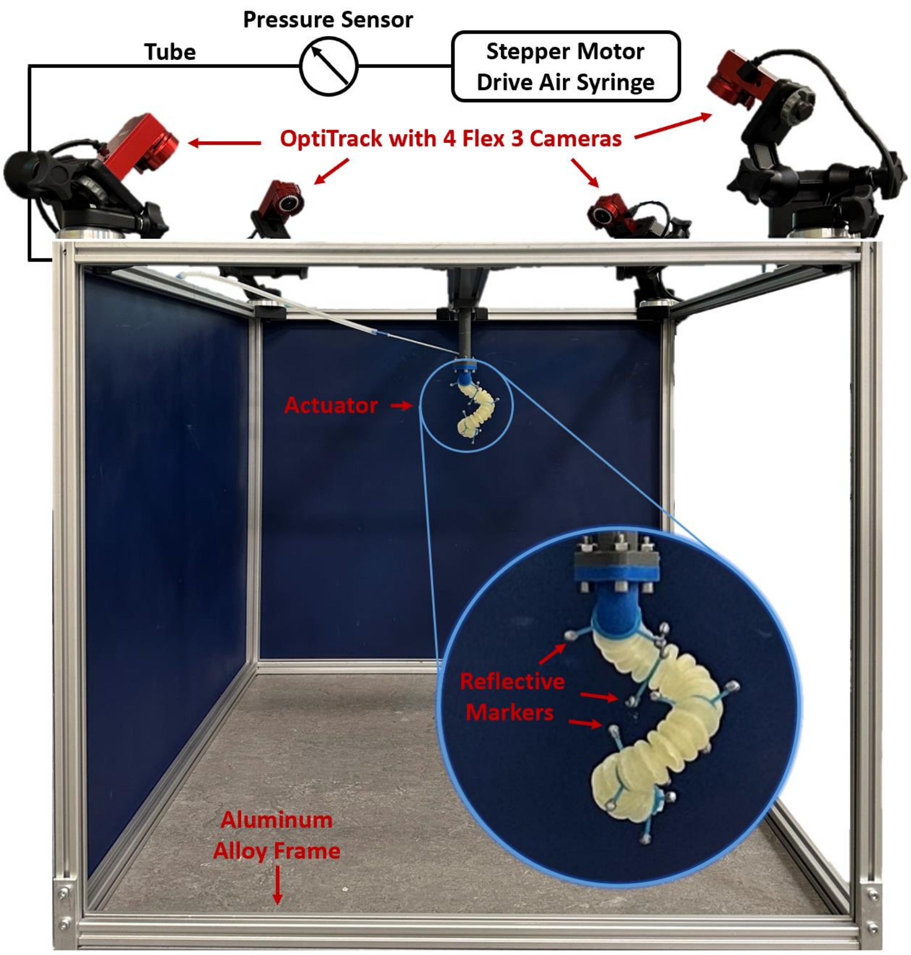


Fig.S7: The experiment setup for the elephant-trunk-inspired, helical-shaped 3D deformable actuator.

The setup of the experiment in Sec.4B is shown in Fig.S7. The top end of the actuator is fixed to a connector (as shown in Fig.7A), which is connected to the top center of a 780×700×563 mm aluminum alloy frame. In order to get the deformed shape of the actuator, we 3D printed five rings, each featuring three evenly spaced rods connected to small reflective tape-coated spheres acting as markers for the OptiTrack™ system. Apart from the actuator's two ends, these rings are situated between every pair of adjacent segments. By identifying the position and orientation of these rings, and presuming a constant curvature between each pair, the real shape of the pressurized actuator can be obtained. The space coordinates of the markers were captured by a motion tracking system (OptiTrack™ with 4 Flex 3 cameras) and can be used to create a rigid body to represent the real-time positions of the free end.

A pneumatic system consisting of a 12V DC stepper motor driving a 60mL air syringe, a micro-controller (Arduino Uno), a pressure sensor (ADP51A11, pressure range 0-40 kPa, Panasonic, Japan) and a PI controller was also used to control the input pressure. We repeated the experiment 3 times for each trial. To analyze the data, we (1) calculated the average positions and orientations of all rings, which were treated as rigid bodies in the OptiTrack™ system, (2) determined arc planes and centers adjacent rings using their positions and tangent vectors, based on the constant curvature assumption, (3) aligned the position and orientation of the top ring with the uppermost point of the desired helical shape, (4) resampled the desired helical shape at the same interval as the sampled arcs' coordinates to derive RMSEs, comparing the desired and real shapes.

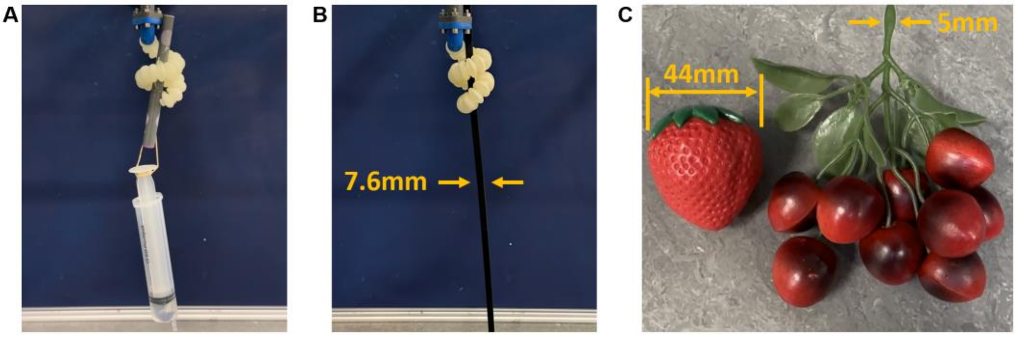


Fig.S8: The supplemental objects for the grasping experiments: A) The object 1 in Fig. 7 carrying a syringe with water. B) A zip tie with a width of 7.6mm. C) Plastic strawberry with a diameter of 44mm and cherry with a handle of 5mm width.

To exploit grasping behaviors arising from the actuator design and interaction with the object, we performed grasping experiments on a series of objects. The object 1 in Fig.7E -- a pen with a diameter of 16mm and length of 120mm, was the motivation for determining the helical shape's radius $a = \frac{16}{2} + r_{ou} = 8 + 10 = 18mm$ and $h = 60mm$ to ensuring a power grasp. The experiment shows that the designed actuator can achieve a successful grasp for this object, which demonstrates the efficiency of our design pipeline. Subsequently, as shown in Fig.S8(A), we attached a 60mL syringe beneath the object and progressively increased its weight by filling it with water to explore the actuator's maximum grasping capacity. We discovered that when the syringe draws in water exceeding 30mL, equivalent to 73g (including the object), the grasp becomes unsuccessful. This is because grasping a heavier object requires higher pressure, which the designed actuator cannot

handle for weights above 73g. Next, we evaluated the actuator's capability using another pen with smaller diameter shown in Fig.7E to assess its ability in grasping objects of reduced diameter. Following a successful grasp, we further tested its capability with even finer objects, confirming its ability to securely grasp items as slender as a zip tie shown in Fig.S8(B). Aside from power grasping, we also explored the actuator's other grasping modes with object 3 and 4, as well as those shown in Fig.S8(C). Object 3 -- a plastic banana represents a grasping mode where only a small part of the object, having a diameter within the preset range, is grasped. Despite the successful grasp of object 3, our attempt with a plastic cherry that has a slimmer handle, as seen in Fig.S8(C), was unsuccessful. This suggests that this grasping mode of the designed actuator has a diameter requirement for the object's contact region. Object 4 -- a tape, represents another grasping mode where the object's geometry is not in the preset range as outlined in Fig.7A. However, the actuator, drawing inspiration from an elephant's trunk, coupled with the tape's mere 8mm thickness, could navigate through the tape's central hole and pinch it effectively. We attempted to use the pinch mode for grasping a plastic strawberry with diameter of 44mm but failed, indicating this grasping model of the designed actuator also has a requirement for objects' size.

In general, an actuator's grasping capability is influenced by its shape, material, and actuation pressure. Utilizing our toolbox necessitates a user-guided method to determine these aspects. By conducting grasping experiments and progressive refinements, users can achieve specific grasping performances. This iterative approach is also adaptable to various other applications.
